# Trim28 acts as restriction factor of prototype foamy virus replication by modulating H3K9me3 marks and destabilizing the viral transactivator Tas

**DOI:** 10.1186/s12977-021-00584-y

**Published:** 2021-12-13

**Authors:** Peipei Yuan, Jun Yan, Shuang Wang, Yang Guo, Xueyan Xi, Song Han, Jun Yin, Biwen Peng, Xiaohua He, Jochen Bodem, Wanhong Liu

**Affiliations:** 1 Department of Immunology, School of Basic Medical Sciences, Hubei University of Medicine, Shiyan, 442000 China; 2 Hubei Province Key Laboratory of Allergy and Immunology, School of Basic Medical Sciences, Wuhan University, No. 185, Donghu Road, Wuchang District, Wuhan, 430071 China; 3 Hubei Provincial Key Laboratory of Developmentally Originated Disease, School of Basic Medical Sciences, Wuhan University, Wuhan, 430071 China; 4 Hubei Key Laboratory of Embryonic Stem Cell Research, Hubei University of Medicine, Shiyan, 442000 Hubei China; 5 Institut für Virologie und Immunbiologie, Julius-Maximilians-Universität Würzburg, 97078 Würzburg, Germany

**Keywords:** Prototype foamy virus (PFV), Trim28, H3K9me3, LTR, Tas

## Abstract

**Background:**

Prototype foamy virus (PFV) is nonpathogenic complex retroviruses that express a transcriptional transactivator Tas, which is essential for the activity of viral long terminal repeat (LTR) promoter and internal promoter (IP). Tripartite motif-containing protein 28 (Trim28) is well known as a scaffold protein normally enriched in gene promoter region to repress transcription. We sought to determine if whether Trim28 could be enriched in PFV promoter region to participate the establishment of PFV latency infection.

**Results:**

In this study, we show that Trim28 restricts Tas-dependent transactivation activity of PFV promoter and negatively regulates PFV replication. Trim28 was found to be enriched in LTR instead of IP promoter regions of PFV genome and contribute to the maintenance of histone H3K9me3 marks on the LTR promoter. Furthermore, Trim28 interacts with Tas and colocalizes with Tas in the nucleus. Besides, we found that Trim28, an E3 ubiquitin ligase, binds directly to and promotes Tas for ubiquitination and degradation. And the RBCC domain of Trim28 is required for the ubiquitination and degradation of Tas.

**Conclusions:**

Collectively, our findings not only identify a host factor Trim28 negatively inhibits PFV replication by acting as transcriptional restriction factor enriched in viral LTR promoter through modulating H3K9me3 mark here, but also reveal that Trim28 mediated ubiquitin proteasome degradation of Tas as a mechanism underlying Trim28 restricts Tas-dependent transcription activity of PFV promoter and PFV replication. These findings provide new insights into the process of PFV latency establishment.

**Graphical Abstract:**

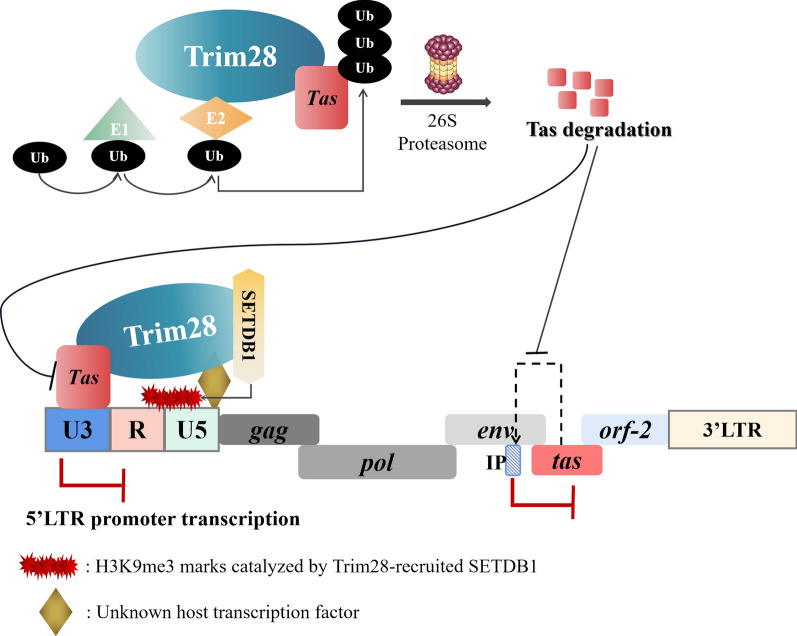

## Background

Prototype foamy virus (PFV) belongs to the group of complex retroviruses, since it encodes the APOBEC3 antagonizing factor Bet and a transcriptional transactivator Tas [1–3]. These regulatory genes are expressed from an internal promoter (IP) localized in the *env* gene [4, 5], while the genomic RNA, the *pol* and *env* transcripts are expressed from the LTR promoter. However, foamy viral gene expression is distinct from other retroviruses in many respects [2, 3]. Tas is a DNA binding protein, which activates both promoters by binding to Tas responsive elements (TREs) upstream of the respective transcriptional start-site [6–8]. While the IP has a moderate basal activity independent of Tas, the activity of LTR promoter is strictly dependent on Tas. The basal activity and the higher affinity of Tas to the IP TREs has led to the hypotheses that the foamy viral gene expression is orchestrated by Tas in an early and a late phase [9]. Furthermore, it has been shown that foamy viruses (FVs) persist in infected animals and accidentally infect humans, which supports a model of FV latency as well. Recently, we have reported that an autophagy process could be induced by PFV infection, which participates in regulating PFV replication [10]. In addition, host factor Pirh2 (human p53-induced RING-H2 protein) and TBC1D16 have also been identified to repress PFV replication [11, 12]. However, the underlying mechanism of PFV latent infection remains elusive.

Trim28, which is also known as KRAB-associated protein 1 (KAP1) and transcription intermediary factor 1β (TIF1β), is a member of the tripartite motif-containing protein (TRIM) family [13]. Trim28 is implicated in a variety of cellular functions such as cell growth, differentiation, oncogenesis, inflammation, apoptosis, autophagy and innate antiviral immunity [14–16]. Trim28 is best known as a prominent scaffold protein mediator of gene silencing, tethered to target DNA by KRAB (Krüppel-associated box) or non-KRAB zinc finger proteins to form a transcription silencing complex to repress downstream gene [17]. It is well known that Trim28 functions as a transcriptional repressor and can change the epigenetic state by recruiting the histone deacetylase complex NuRD (Nucleosome Remodeling Deacetylase), histone H3 lysine 9 specific methyltransferase SETDB1 (SET domain bifurcated 1, also called ESET or KMT1E) and HP1 (heterochromatin protein 1) [18, 19]. The repression mediated by the Trim28 complex can exert a long-range effect on the genome by spreading SETDB1-catalyzed histone H3 lysine 9 trimethylation (H3K9me3) to play important roles in silencing of genes and retroelements [20]. This negative role of Trim28 on gene transcription has important implications in silencing viral transcription and replication. It was shown that Trim28 restricts murine leukaemia virus (MLV) replication in embryonic carcinoma and embryonic stem cells (ESCs) [21, 22]. Furthermore, Trim28, together with H3K9me3 methyltransferase, SETDB1 and HP1, mediate the silencing of endogenous retroviruses (ERVs) in embryonic stem cells and in neural progenitor cells [23, 24]. Trim28 was reported to restrict human immunodeficiency virus type 1 (HIV-1) replication by binding the acetylated HIV-1 integrase and to hinder integration of the proviral DNA [25]. It can also mediate the transcription repression of HIV-1 LTR promoter [26, 27]. In addition, Trim28 complex has been shown to repress expression of episomal gene expression, such as adeno-associated viral (AAV) vectors and integration-defective lentiviral vectors (IDLV) [28].

However, the effect of Trim28 on regulating PFV latency has not been investigated so far. Here we report that Trim28 restricts PFV transcription and replication by inhibiting Tas-dependent transactivation activity of PFV promoters. We observed that Trim28 is highly enriched in viral LTR promoter regions to maintain H3K9me3 marks here. Furthermore, Trim28 binds to the viral Tas protein, destabilizing Tas in ubiquitination pathway, therefore disturbing the transactivation function of Tas. Our results revealed the mechanism of negatively regulation by Trim28 in PFV replication, providing new insights into the process of PFV latency establishment.

## Results

### Trim28 inhibits PFV replication and transcription

**Fig. 1:**
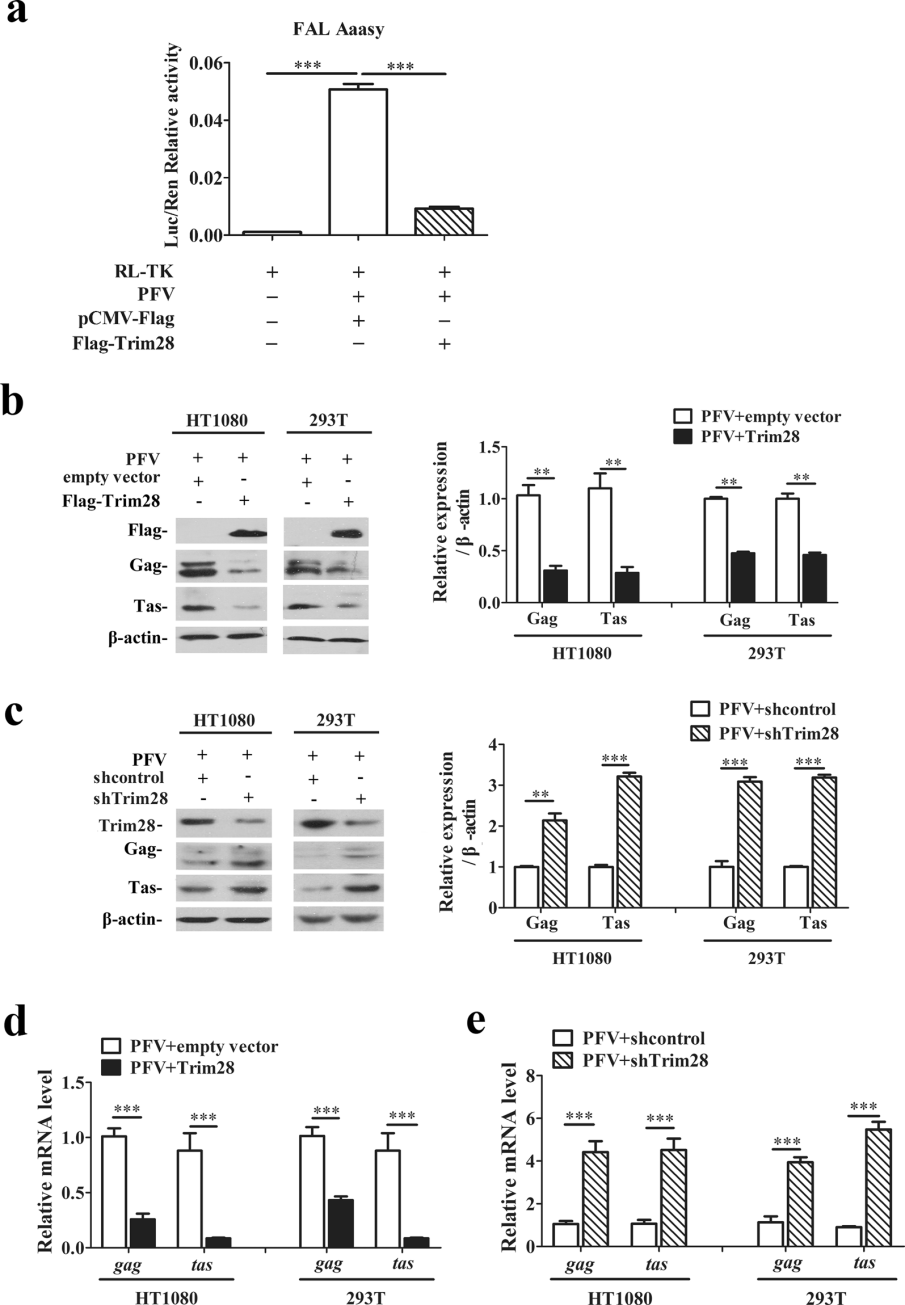
Trim28 inhibits prototype foamy virus replication. **a** The relative viral load in the presence or absence of overexpressed Trim28 was analyzed in PFV indicator cell line using the FAL assay. RL-TK (5 μg) was transfected as an internal control. **b** HT1080 and HEK293T cells were transfected with pCMV-Flag-Trim28 or pCMV-Flag (as an empty control) for 24 h. After transfection, cells were infected with PFV (MOI = 0.1) for 48 h. The expression of PFV Gag and Tas were detected by Western blot. **c** Specific shRNAs was used to knockdown Trim28 and the shControl was used as negative control. The viral proteins Gag and Tas were detected by Western blot. Quantitation analysis of Gag and Tas intensity from the western blotting using Quantity one software (Bio-Rad). **d** Relative mRNA expression (normalized to β-actin) of the viral structural gene *gag* and of all genes encompassing the *tas* region in the PFV-infected HEK293T or HT1080 cells that were transfected with pCMV-Flag-Trim28 or pCMV-Flag (as an empty control) for 24 h. **e** Relative mRNA expression (normalized to β-actin) of the viral structural gene *gag* and of all genes encompassing the *tas* region in the PFV-infected HEK293T or HT1080 cells that were transfected with shTrim28 or shControl were assessed by qPCR. All the data are representative of three independent experiments with triplicate samples. (paired *t-*test; *p < 0.05, **p < 0.01, ***p < *0.001*)

To investigate the effect of Trim28 on PFV replication, we first used a PFV indicator cell line (PIC) in which a luciferase gene driven by the PFV LTR promoter was stably transfected into baby hamster kidney-21 (BHK-21) cells. Since the activity of LTR is strictly dependent on Tas, the luciferase gene is only expressed when Tas is present in the system, and the expression level is directly proportional to the amount of Tas. Therefore, it can be used to measure virus titer of PFV and is more sensitive than TCID50 [29]. In this foamy virus activated luciferase (FAL) assay, Flag-Trim28 were transfected into HEK293T cells for 24 h, pCMV-Flag was transfected as a control, and the cells were challenged with PFV at a multiplicity of infection (MOI) of 0.1 for another 48 h, then those infected HEK293T were incubated with PIC for 48 h, and RL-TK plasmid expressing Renilla luciferase was transfected into PIC as an internal control 12 h before incubation. The FAL assay results showed that PFV was able to be more prominently activated the PIC compared to the control group, and the Luc/Rlu ratio of Flag-Trim28-transfected cells was significantly reduced compared to the control group (Fig. 1a), suggesting that overexpression of Trim28 significantly suppressed PFV viral load. To further analyze the effects of Trim28 on viral proteins production, the levels of PFV viral protein Gag (3.3-fold change in HT1080 cells, 2.1-fold change in 293T cells) and Tas (3.8-fold change in HT1080 cells, 2.2-fold change in 293T cells) were analyzed by quantitative western blotting and found to be modest downregulated by overexpression of Trim28 in a cell type independent way (Fig. 1b). In addition, Trim28 expression was downregulated by transfection specific Trim28-shRNA plasmid (Fig. 1c). After transfection with Trim28-shRNA plasmid, cells were infected with PFV (MOI = 0.1) for 48 h. Compared with the control group, both the protein expression of Gag (2.1-fold change in HT1080 cells, 3.1-fold change in 293 T cells) and Tas (3.2-fold change in HT1080 cells or in 293T cells) were elevated upon Trim28 knockdown (Fig. 1c). To further explore whether Trim28 suppresses viral transcription, the relative RNA amounts of all viral RNA, primers located in the Tas region, or of the LTR derived genomic RNA were analysed by RTqPCR from PFV infected cells, which either overexpression of Trim28 or transfected with the Trim28 specific shRNA (Fig. 1d and e). Amounts of all viral transcripts containing the *tas* region (*tas* group, tenfold change in HT1080 or 293T cells) and of the genomic RNA (*gag* group, 3.8-fold change in HT1080 and 2.3-fold change in 293T cells) were decreased in cells overexpressing Trim28 compared to the empty control group (Fig. 1d). In line with this finding, quantification of all viral transcripts and of the genomic RNA of PFV in cells transfected with Trim28 specific shRNA showed a more than two-fold increase (Fig. 1e). The formation of PFV proviruses is established within 12 h post-infection [30], thus the effect of the viral genomic RNA or viral protein detected above representing the viral life cycle stage with formation of a stable DNA form after integrated into host genome. These results suggested that Trim28 could inhibit PFV replication, and also could repress viral transcription already in the early stage of viral life cycle.

### Trim28 is enriched in the PFV LTR promoter

**Fig. 2:**
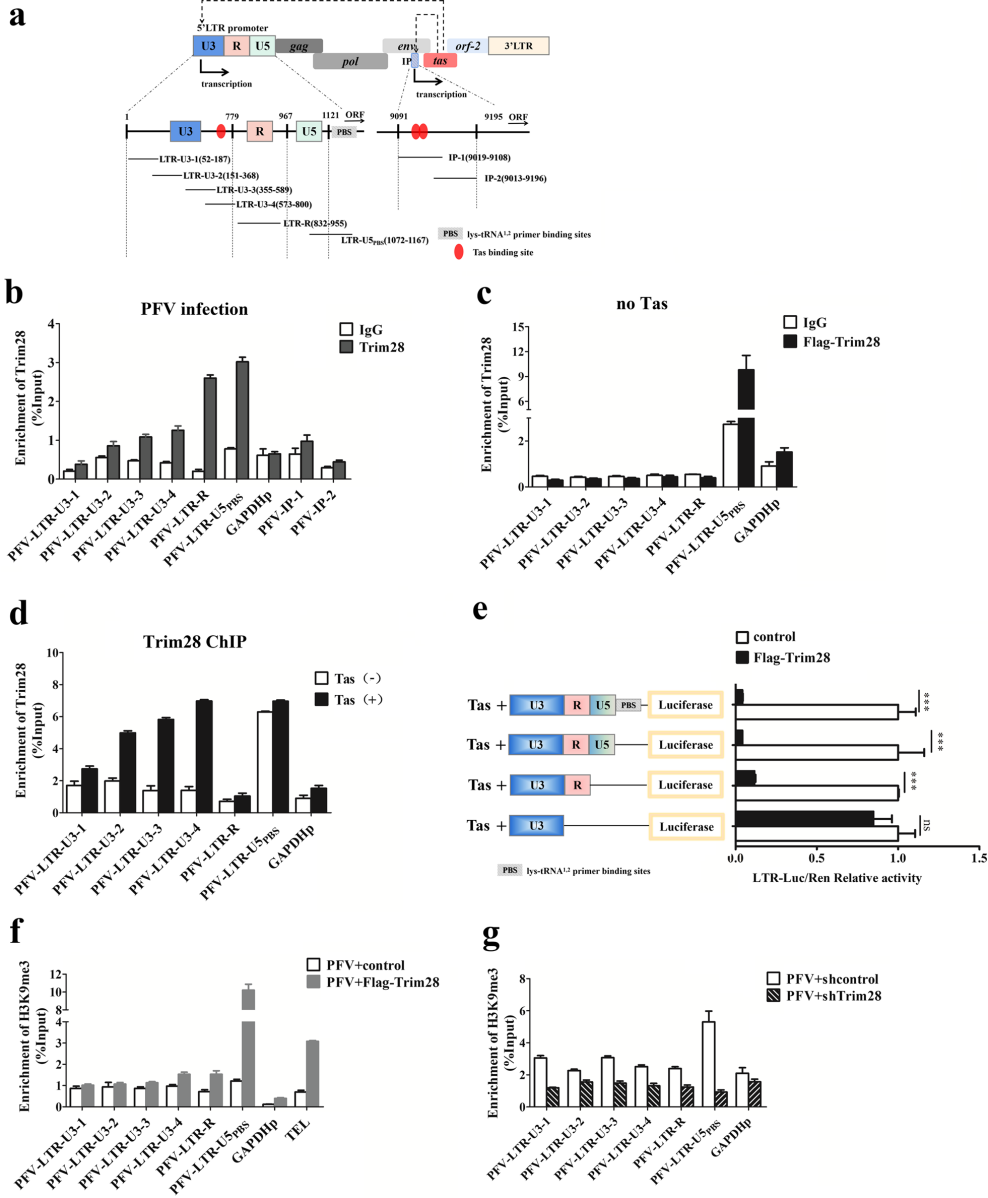
Trim28 is enriched in PFV 5′LTR region. **a** Illustration of the primer sets at the PFV LTR and IP promoter used in the ChIP assays. The numbers are relative to the transcription start site nucleotide+1. **b** Trim28 was enriched in the PFV 5′LTR regions during PFV infection. ChIP assay was performed on cells infected with PFV for 48 h using IgG, or anti-Trim28 Ab. ChIP-qPCR data were normalized by the percent input method (%input with IgG as control) with GAPDH promoter region as the negative control*.* The data presented are means the standard errors of the means of three independent experiments. **c** Trim28 is enriched in PFV 5′LTR U5 region in the absence of Tas. Trim28 was enriched in the PFV 5′LTR regions. ChIP assay was performed on PFV-5′LTR-transfected cells using IgG, or anti-Trim28 antibody. ChIP-qPCR data were normalized by the percent input method (%input with IgG as control) with GAPDH promoter region as the negative control. The data presented are means the standard errors of the means of three independent experiments. **d** ChIP assays of Trim28 in the presence (+) or absence (−) of Tas expression. Plasmid pGL3-basic-PFV-LTR containing the LTR promoter was transfected into cells with Tas expression plasmid or empty-vector controls. Cells were collected for ChIP assay at 24 h post-transfection. 8 h before harvest, the cells were treated with MG132 (5 μM). ChIP-qPCR data were normalized by the fold enrichment method (ChIP signals were divided by the IgG signals) with GAPDH promoter region as the negative control. **e** The effect of Trim28 in regulating the activity of Tas-dependent transactivation of the PFV LTR and truncated-LTR promoter activity. Schematic represent of the PFV LTR or truncated-LTR-driven expression of firefly luciferase. HEK293T cells seeded in 24-well plates were transfected with pCMV-Flag or pCMV-Flag-Trim28 (400 ng), pRL-TK (3 ng), pTK-Tas (50 ng) and truncated pGL3-PFV-LTR-luc (70 ng) firefly luciferase reporter. Luciferase activities were measured as described in the Materials and Methods. (paired *t-*test; * p < 0.05, ** p < 0.01, *** p < *0.001*). **f**, **g** Trim28 promotes H3K9me3 recruiting to the PFV 5′LTR regions. ChIP assays of H3K9me3 with the overexpression or knockdown of the Trim28 expression in PFV infected HEK293T cells. ChIP-qPCR data were normalized by the fold enrichment method (ChIP signals were divided by the IgG signals). The data are presented as the means ± SD. The telomere (Tel) genomic region acted as a positive control, and GAPDH promoter region acted as a negative control when Trim28 was overexpressed during PFV infection

Previous studies showed that Trim28 could be recruited to endogenous retroviral or retroviral lys-tRNA^1,2^ primer binding sites (PBS) by KRAB-zinc fingers and other factors to repress transcription from the viral promoter [23]. There is also a lys-tRNA^1,2^ primer binding site at the 3′boundary of PFV 5′ LTR promoter [31]. We assumed that enrichment of Trim28 in the PFV LTR promoter would inhibit LTR promoter transcription. It is universally known that the chromatin immunoprecipitation (ChIP) assay is an effective method to study protein-gene interactions in vivo [32]. We detected the enrichment of Trim28 in the PFV LTR and IP promoter regions by ChIP assay with anti-Trim28 and the control IgG antibodies. To explore whether Trim28 could enrich in the PFV LTR and IP promoters, we designed six pairs of primers at the different regions of LTR promoter and two pairs of IP primers to determine the Trim28 enrichment site in ChIP assay (Fig. 2a). HT1080 cells were infected with PFV and collected for ChIP assay with anti-Trim28 antibody 48 h later. The purified DNA eluate was quantified by qPCR (Fig. 2b). Interestingly, we found that endogenous Trim28 was enriched in the LTR regions, especially significantly enriched in R (tenfold change, p < 0.01), U5 and downstream in the PBS regions (threefold change, p < 0.01) (Fig. 2b). These ChIP experiments were repeated three times independently. For IP regions, Trim28 was hardly enriched in it (Fig. 2b). To further study the enrichment of Trim28 in the LTR promoter when viral protein Tas is absence, we ultimate ChIP assay in which HT1080 cells were con-transfected with pGL3-basic-LTR and Flag-Trim28 (with no Tas expression in the system) for 48 h. Then anti-Flag antibody was used for detected the enrichment of Flag-Trim28 in LTR promoter with no Tas. We found that Flag-Trim28 was only highly enriched in U5-PBS regions in LTR promoter with no Tas expression in the system (Fig. 2c). To demonstrate the role of Tas in Trim28 recruitment, that is, whether the recruitment of Trim28 to the LTR promoter regions was dependent on Tas, we determined the binding of Trim28 in the presence or absence of Tas expression. Plasmid pGL3-basic-LTR, containing the LTR promoter, was transfected into cells with or without Tas expression plasmid. Cells were collected for ChIP assay at 24 h post-transfection and treated with MG132 before harvest for ChIP assay. Figure 2d shows that enrichment of Trim28 in the LTR promoter region was detected only in U5-PBS region in cells with no Tas expression (−). However, in the presence of Tas (+), Trim28 was enriched in the LTR regions, especially enriched in U3, U5 and downstream in the PBS regions (Fig. 2d). In order to verify these results, we constructed a series of truncated LTR reporter plasmids to define the Trim28 responsive region functionally. Overexpression of Trim28 inhibited Tas-dependent PFV LTR promoter activity in constructs encompassing the R, U5 and PBS regions, whereas, it did not affect the promoter activity of with U3 region alone (Fig. 2e).

Previous studies have shown that Trim28 induces repressive posttranscriptional modifications by recruiting multi-protein complexes including the H3K9 methyltransferase SETDB1 [18, 19]. Therefore, we sought to analyze, whether repressive H3K9me3 mark was enriched in the PFV LTR regions by ChIP assay with anti-H3K9me3 antibody in PFV-infected cells overexpressing Trim28. Cells were collected for ChIP assay at 48 h post infection. Indeed, H3K9me3 was found to be recruited to the adjacent to R (U3-4), R, and U5 regions of LTR promoter and PBS regions in PFV-infected cells with overexpression of Trim28, especially highly enriched in U5 region (Fig. 2f). Conversely, the enrichment of H3K9me3 in the PFV LTR promoter was decreased in PFV-infected HT1080 cells after knockdown of Trim28 (Fig. 2g). These results demonstrated that Trim28 was enriched in PFV 5′LTR region but not in IP region. In addition, H3K9me3 which was catalyzed by Trim28-recruited SETDB1, were also enriched in PFV 5′LTR region. All together, these results implied that Trim28 could enrich in PFV LTR promoter regions to negatively regulate PFV LTR promoter activity. Tas might influence the enrichment region of Trim28 in PFV LTR promoter. Furthermore, we collected evidence that Trim28 could contribute to maintain H3K9me3 marks in the PFV LTR promoter region.

### Trim28 negatively regulates Tas-dependent transactivation of PFV LTR and IP promoter

**Fig. 3:**
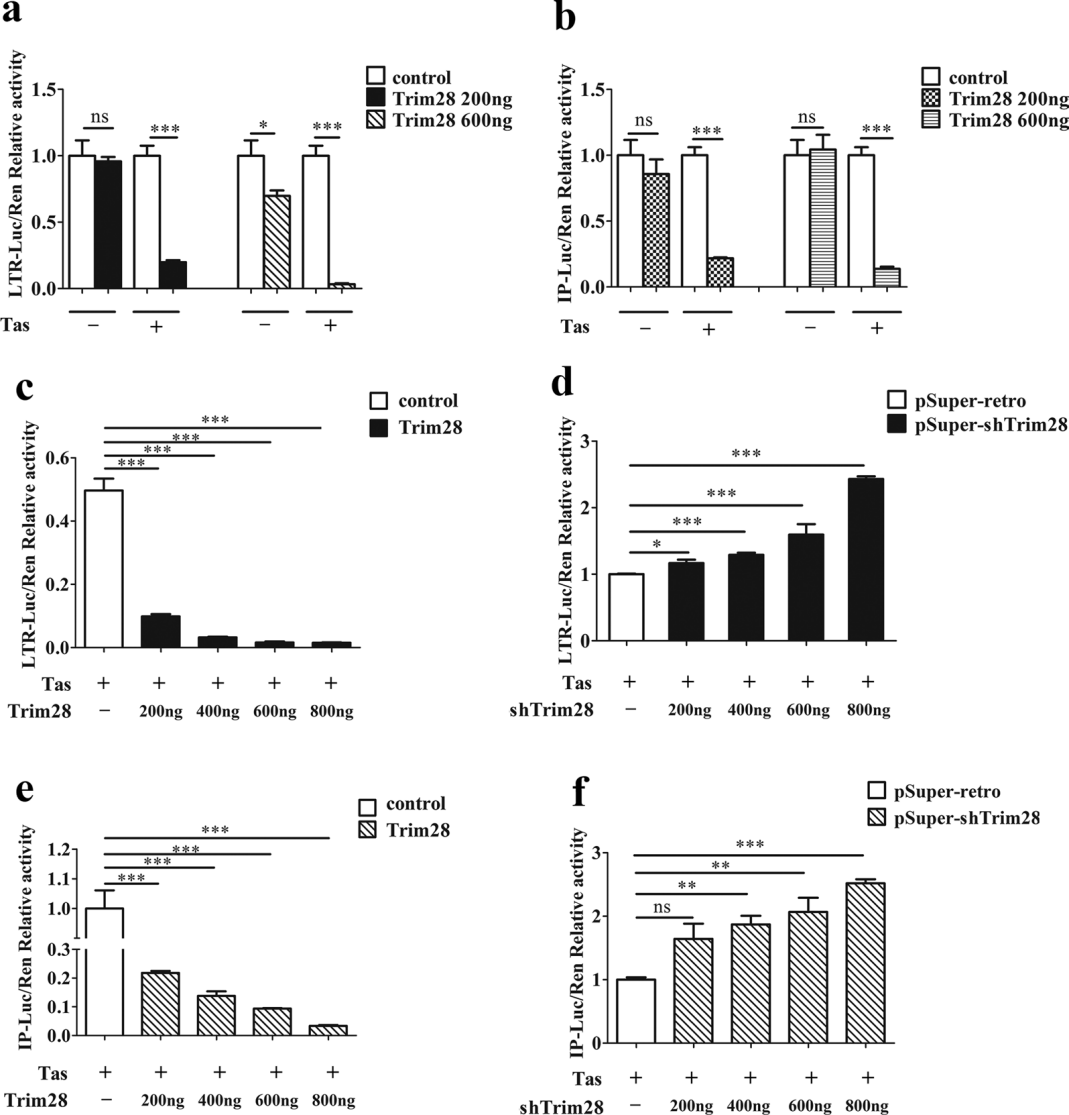
Trim28 negatively regulates Tas-dependent transcriptional activation of PFV LTR and IP promoter activity. **a** Trim28 inhibited the Tas-dependent transactivation activity of PFV LTR promoter. HEK293T cells seeded in 24-well plates were co-transfected with pCMV-Flag-Trim28 (200 ng or 400 ng, and pCMV-Flag as an empty control), pRL-TK (3 ng), and pGL3-PFV-LTR-luc (70 ng) firefly luciferase reporter. These co-transfected plasmids were combined with or without pTK-Tas (50 ng). **b** Trim28 inhibited the Tas-dependent transactivation activity of PFV IP promoter. HEK293T cells seeded in 24-well plates were transfected with pCMV-Flag or pCMV-Flag-Trim28 (200 ng or 400 ng), pRL-TK (3 ng), with or without pTK-Tas (20 ng) and pGL3-PFV-IP-luc (20 ng) firefly luciferase reporter. **c** Trim28 inhibited the Tas-dependent transactivation activity of PFV LTR promoter in a dose dependent way. HEK293T cells seeded in 24-well plates were transfected with pCMV-Flag or pCMV-Flag-Trim28 (0 ~ 800 ng), combined with pRL-TK (3 ng), pTK-Tas (50 ng) and pGL3-PFV-LTR-luc (70 ng) firefly luciferase reporter. **d** Knockdown of Trim28 with its specific shRNA up-regulates Tas-dependent transactivation of the PFV LTR promoter a dose dependent way. HEK293T cells seeded in 24-well plates were first transfected with pSuper-shNC or pSuper-shTrim28 (0 ~ 800 ng) for 24 h and then cells were cotransfected with pRL-TK (3 ng), pTK-Tas (50 ng) and pPFV-LTR-luc (70 ng) firefly luciferase reporter for 24 h. **e** Trim28 inhibited the Tas-dependent transactivation activity of PFV IP promoter in a dose dependent way. HEK293T cells seeded in 24-well plates were transfected with pCMV-Flag or pCMV-Flag-Trim28 (0 ~ 800 ng), pRL-TK (3 ng), pTK-Tas (20 ng) and pGL3-PFV-IP-luc (20 ng) firefly luciferase reporter. **f** Knockdown of Trim28 with its specific shRNA up-regulates Tas-dependent transactivation of the PFV IP promoter a dose dependent way. HEK293T cells seeded in 24-well plates were first transfected with pSuper-shNC or pSuper-shTrim28 (0 ~ 800 ng) for 24 h and then cells were cotransfected with pRL-TK (3 ng), pTK-Tas (20 ng) and pGL3-PFV-IP-luc (20 ng) firefly luciferase reporter. Luciferase activities were measured as described in the Methods. (paired *t-*test; *p < 0.05, **p < 0.01, ***p < *0.001*)

LTR and IP are key promoters to regulating PFV genome transcription and determining whether the infection is lytic or persistent [33]. IP has modest basal activity without Tas and it drives initial Tas expression in the initial stage of PFV replication. After being expressed, Tas transactivates LTR and IP promoters to initiate virus replication [34]. To analyze the function of the highly enrichment of Trim28 in the LTR promoter, the influences on LTR promoter activity were measured by luciferase reporter gene assay [2, 35]. Meanwhile we detected its influences on IP promoter transcription activity, although Trim28 was hardly enriched in IP promoter which was shown in previous results (Fig. 2b). HEK293T cells were co-transfected with LTR-Luc (pGL3-PFV-LTR-luc) or IP-Luc (pGL3-PFV-IP-luc) reporter plasmids with a Tas-expression vector and with increasing amounts of Trim28-expression vector. A Renilla expression plasmid was co-transfected to allow normalization of transfection efficiencies. Luciferase activities were measured 24 h post-transfection. Overexpression of Trim28 potently reduced Tas-dependent transactivation of LTR and IP promoter activity in a dose-dependent manner (Fig. 3c and e). In contrast, overexpression of Trim28 slightly inhibited the basal transcription activity of LTR promoter in absence of Tas (with no Tas expression), and almost no effect on the basal activity of the IP (Fig. 3a and b). In line with the dose-dependent repression of Tas-dependent transactivation, Trim28-specific shRNA transfection resulted in elevated Tas-dependent transactivation of PFV LTR and IP promoter activities compared to the controls (Fig. 3d and f). These results indicated that Trim28 negatively regulates Tas-dependent transcription of both PFV promoters. In addition, combined with the previous result that Trim28 is highly enriched in LTR but not IP promoter, however, it could inhibit PFV LTR and IP promoter in Tas-dependent way. We assumed that Trim28 might interact with Tas in cells to influence its transactivation function.

### Trim28 interacts with Tas in vivo

**Fig. 4:**
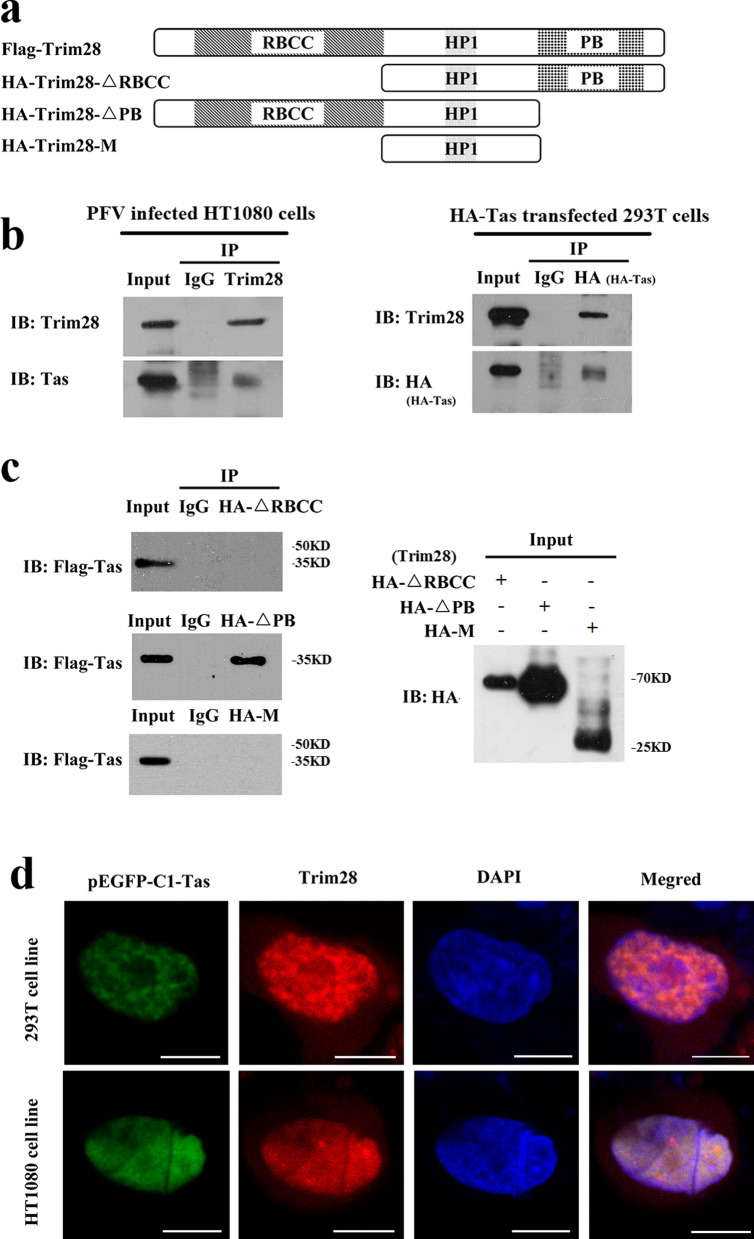
Trim28 interacts with Tas in vivo. **a** Scheme of Trim28 domains and truncated constructs used in this study. RBCC domain represent ring finger, B-box, zinc finger, coiled-coil domain at its amino-terminus; PB is a planthomeodomain and bromodomain at its carboxyl-terminus; and a domain for HP1 binding in the middle of entire gene. **b** Trim28 interacts with Tas in vivo. HT1080 cells were infected with PFV for 48 hpi. Forty-eight hours later, the infected HT1080 cells were lysed, and immunoprecipitation was performed with a negative control IgG or anti-Trim28 antibody. Then, the immunoprecipitates were detected by Western blot using anti-Trim28 and anti-Tas antibodies. HEK293T cells were co-transfected with Flag-Trim28 and HA-Tas. Forty-eight hours post transfection, the whole cell lysate was harvested and Tas was immunoprecipitated with anti-HA and normal IgG, which was used as negative control. Then, the immunoprecipitates were detected by western blotting using anti-HA and anti-Flag antibodies. **c** The RBCC domain of Trim28 interacts with Tas in vivo. The HEK293T cells were co-transfected with HA-Trim28-△RBCC/Trim28-△PB/Trim28-M and Flag-Tas, and 48 h post transfection, the whole cell lysate was harvested and immunoprecipitated with anti-HA and normal IgG, which was used as negative control. Then, the immunoprecipitates were detected by Western blot using anti-Flag, and the input sample were detected by Western blot using anti-HA antibody. **d** Tas was colocalized with Trim28 in the nucleus in vivo. pEGFP-N1-Tas was transfected into HT1080 or HEK293T cells, and 48 h after transfection, Trim28 was visualized with anti-Trim28, while the nuclei were visualized with DAPI staining. A merged image is shown on the bottom. Scale bar, 7.5 μm

To analyze the mechanism of the inhibition of PFV 5′LTR and IP in Tas-dependent transactivation and to assess the interplay between Trim28 and Tas in vivo, co-immunoprecipitation experiments were performed. HT1080 cells were infected with PFV for 48 hpi following immunoprecipitation with Trim28 specific antibodies. The precipitates were assessed by western blotting using anti-Tas and anti-Trim28 antibodies and showed that Tas was co-immunoprecipitated with endogenous Trim28 in HT1080 cells (Fig. 4b). Similarly, Trim28 was co-immunoprecipitated with HA-tagged Tas (Fig. 4b). As a member of the TRIM protein family with typical structural features [13], Trim28 contains a ring finger, B-box, zinc finger, coiled-coil (RBCC) domain at its amino-terminus; a planthomeodomain and bromodomain at its carboxyl-terminus; and a domain for HP1 binding in the middle of entire gene (Fig. 4a). To determine the interaction domain of Tas and Trim28, HEK293T cells were co-transfected with a series of HA-tagged truncated forms of Trim28 and with Flag-tagged Tas (Fig. 4a). Only the interaction of Trim28-△PB with Flag-tagged Tas could be shown, while Trim28-△RBCC or Trim28-M failed to interact with Tas indicating that Tas interacted with wild-type or Trim28 mutant lacking the PB domain, but not with Trim28 lacking the RBCC domain (Fig. 4c). Furthermore, Tas did not associate with the HP1 domain of Trim28 (Fig. 4c) suggesting that the interaction between Trim28 and Tas is independent of the PB domain of Trim28, and N-terminal domain of Trim28 containing RBCC is critical for Tas binding. Then the interaction of Trim28 and Tas was confirmed by indirect immunofluorescence using confocal microscopy. HT1080 and HEK293T cells were transfected with a GFP-tagged Tas (pEGFP-N1-Tas) expression plasmid. Trim28 localization was determined with an anti-Trim28 antibody. In support of our co-immunoprecipitation data, the confocal microscope analyses showed that pEGFP-N1-Tas and endogenous Trim28 colocalized within the nucleus in HT1080 and in HEK293T cells (Fig. 4d). These results indicate that Tas interacts with Trim28 in the nucleus and the RBCC domain of Trim28 might be the functional region for their interaction.

### Trim28 destabilizes Tas through ubiquitination

**Fig. 5:**
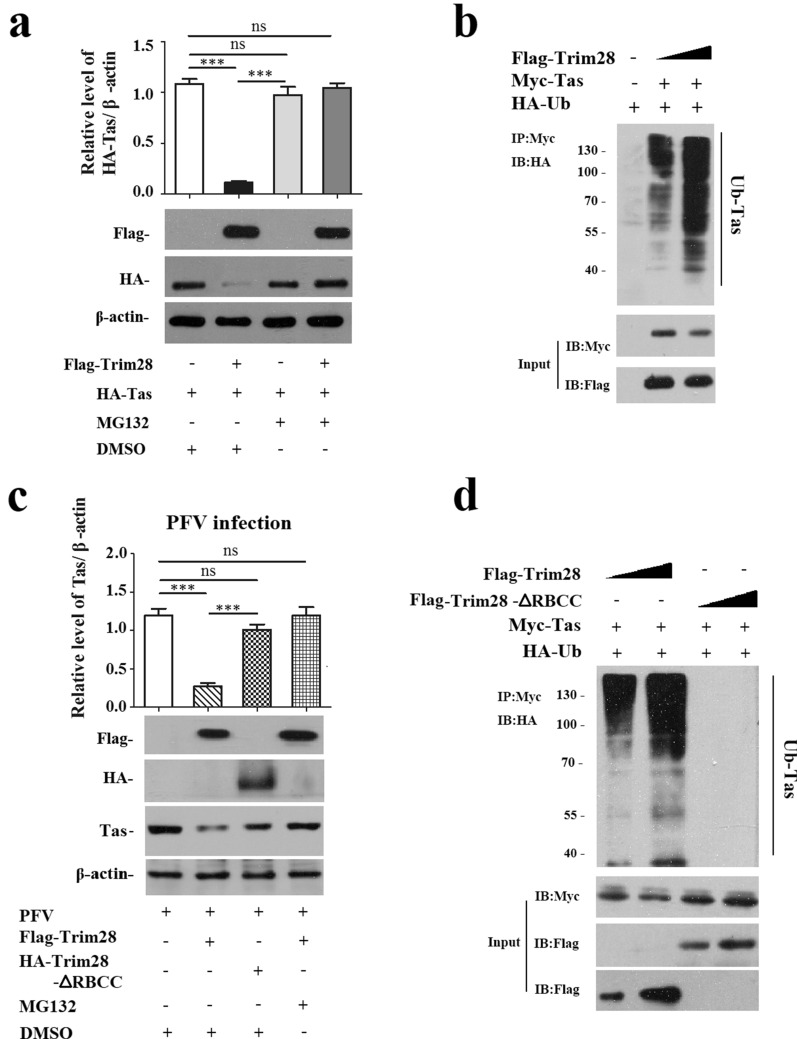
Trim28 decreases the expression of Tas protein and inhibits PFV replication through ubiquitin–proteasome pathway. **a** Trim28 down regulates the expression of Tas protein and the inhibition was reversed by MG132. HA-Tas combined with Flag-Trim28 or pCMV-Flag (as control) were transfected into HEK293T cells; 8 h before harvest, the cells were treated with DMSO or MG132 (5 μM). Western blot was performed to assess Tas protein levels; **b** Trim28 promotes Tas polyubiquitination. HA-tagged ubiquitin (HA-Ub), Myc-Tas combined with Flag-Trim28 were transfected into HEK293T cells, pCMV-Flag used as negative control. **c** MG132 rescues the suppression of PFV produced by overexpressed Trim28, and deletion of RBCC domain abrogates its inhibitory effect. HT1080 cells were transfected with Flag-Trim28, HA-Trim28-△RBCC or pCMV-Flag (as control) for 24 h and challenged with PFV (MOI = 0.1) for another 48 h. 8 h before harvest, one group of cells transfected with Flag-Trim28 were treated with MG132 (5 μM, another groups were treated with DMSO). **d** Deletion of RBCC domain of Trim28 cannot cause trigger the ubiquitination of Tas. In cellular ubiquitination assay, HEK293T cells were transfected with HA-tagged ubiquitin (HA-Ub), Myc-Tas combined with Flag-Trim28 or Flag-Trim28-△RBCC, pCMV-Flag used as negative control

Because of the multiple functions of Trim28 participating in post transcriptional modification of interacting factors and the RING finger domain forming a complex with various proteins to target them for ubiquitination degradation [36], we speculated that, as an E3 ubiquitin ligase Trim28 might affect the stability of Tas by ubiquitin pathway [37, 38]. HA-Tas was co-transfected with Flag-Trim28 and pCMV-Flag was used as control. As shown in Fig. 5a, overexpression of Trim28 led to a decline in the level of Tas protein compared to the control. To further confirm whether the molecular mechanism is related to the proteasome degradation pathway, Trim28-overexpressing cells were treated with the proteasome inhibitor MG132 at 5 μM for 8 h before lysis, and we found that Tas expression was notably rescued by MG132 (Fig. 5a), indicating that Trim28 may decrease Tas through the ubiquitin–proteasome pathway. Then, we performed an in vivo ubiquitination assay. The HEK293T cells were transfected with HA-tagged ubiquitin (HA-Ub), Myc-Tas combined with Flag-Trim28, and pCMV-Flag used as a negative control, or Trim28 was transfected with increasing amount as a self control. Immunoprecipitation was used with anti-Myc or anti-Flag antibodies, and the precipitates were analyzed with anti-HA antibody. Overexpression of Trim28 was found to significantly increased Myc-Tas polyubiquitination, which its polyubiquitination was boosted with increased Trim28, indicating that Trim28 polyubiquitinated Tas and mediated its proteasomal degradation (Fig. 5b). The RBCC domain of Trim28 is required for its E3 ligase activity [39]. And we found that the RBCC domain is an important interacting domain for Trim28 and Tas interaction in previous result. We further tested by western blot whether deletion of the RBCC domain would reverse the inhibitory effect of Trim28 on PFV replication. As shown in Fig. 5c, Tas was hardly decreased by Trim28-ΔRBCC compared to Trim28. To determine whether Trim28-ΔRBCC could influence Tas by ubiquitination, we performed an in vivo ubiquitination assay by overexpressing Flag-Trim28-ΔRBCC to assess whether deletion of RBCC domain of Trin28 could trigger the ubiquitination of Tas. Figure 5d shows us that, overexpression of Trim28-ΔRBCC did not give rise to Myc-Tas polyubiquitination compared with overexpression of Trim28, suggesting the RBCC domain is necessary for Trim28 in destabilizing Tas through ubiquitination. All of these results indicate that Trim28 negatively influences viral replication by promoting the instability of Tas through the ubiquitin pathway.

## Discussion

Here, we provided evidence that Trim28 is a restriction factor for prototype foamy viruses, which suppresses viral transcription by destabilizing Tas through ubiquitination and by changes of chromatin state at the LTR promoter. Trim28 has been implicated in the repression of retroviral transcription by being enriched in the LTR promoter region. Nishitsuji et al. showed that Trim28 could be recruited in the HIV-1 LTR promoter by ZBRK1, which in conjunction with Trim28 and HDAC2, suppresses the HIV-1 LTR-driven gene expression [26]. It was reported that the transcriptional activity of the HIV-1 LTR promoter could be repressed by ZNF10 binding with silencing complex containing Trim28, SETDB1 and HP1. These silencing complexes were identified enriched in the HIV-1 LTR promoter [27]. In embryonic cells, Wolf et al. reported that M-MLV and HTLV-integrated proviral DNAs were potently silenced at the transcriptional level, which was largely due to Trim28 binding to the primer binding site (PBS) of the LTR region of M-MLV and HTLV and repressing transcription from the viral LTR promoter [21, 40]. Recently, ZNF304 was been screened out to silence HIV gene transcription through associating with Trim28 and recruiting to the viral promoter heterochromatin-inducing methyltransferases PRC and SETB1 [41]. In the ChIP assay, we demonstrated Trim28 is strong enriched in the LTR promoter but weak enriched in the IP promoter during PFV infection. The enrichment of endogenous Trim28 in the R, U5 and PBS region was stronger than the U3 region of LTR during PFV infection. The recruitment assay demonstrated that transient transfected Tas recruited Trim28 to the LTR promoter region U3 region compared with no Tas expression (Fig. 2d). The enrichment of Trim28 in U5-PBS region is present in transiently transfected cells both in absence and presence of Tas. However, the binding of Trim28 in R region was weak in the cells transiently transfected with LTR promoter plasmid which was different from the observation in PFV-infected cells. The transient transfection could be regarded as a process mimicking PFV primary infection, and thus, the result suggested that Trim28 bound to the U3 and U5-PBS region in PFV LTR promoter but not the R region, at the early stage of PFV primary infection. The binding of Trim28 in the R region might be a later event after the Tas establishment of PFV latency. In addition, we observed that overexpression of Trim28 did not affect Tas-dependent PFV LTR promoter activity with U3 region alone (Fig. 2e). Here, we further illustrated downregulation of the H3K9me3 level in LTR promoter region in PFV-infected cells with knockdown of Trim28, which suggests that Trim28 utilizes inhibitory histone modification H3K9me3 mark to repress PFV gene transcription. And the enrichment of H3K9me3 mark in LTR promoter region in PFV-infected cells with overexpression of Trim28 mainly located in R and U5-PBS region. These results suggested that the inhibitory effect of Trim28 on LTR promoter is also depend on the the epigenetic state of the co-enriched region.

In addition, our results showed that Trim28 inhibits viral promoter transcription dependent on Tas expression suggest that the interaction between Trim28 and Tas is another probable related factors of Trim28 negatively regulating PFV transcription and replication. As a transactivator of PFV, Tas is essential for viral replication and has been proposed to take effect on PFV transcription and replication through different molecular mechanisms. Regad et al. found that promyelocytic leukemia protein (PML) represses PFV transcription by complexing with Tas to prevent its direct binding to the viral DNA [42]. Hu et al. reported that N-Myc interactor (Nmi) can inhibit PFV replication by sequestering Tas in the cytoplasm so that the Tas-mediated transactivation of the viral LTR and IP can be diminished [43]. Previously, we also identified that Pirh2 can interact with Tas and inhibit PFV replication by reducing the Tas protein level through ubiquitin–proteasome pathway [11]. Recently, Kane et al. found that the macaque PHD finger domain protein-11 (PHF11) inhibits basal expression from the IP, thereby preventing Tas expression to inhibit PFV replication [44]. Here, we have shown that Tas interacts with Trim28 in vivo. By studying the enrichment of Trim28 on LTR elements in the presence or absence of Tas, Trim28 was shown to be enriched in the U3 region of LTR promoter in the presence of Tas expression (Fig. 2d). LTR U3 region contains the Tas responsive elements (TREs) for its binding, these results indicated that Tas might be involved in manipulating the interaction between Trim28 and PFV LTR promoter. In absence of Tas expression, Trim28 was also enriched in U5-PBS region of LTR promoter (Fig. 2c and 2d). We suspected that, during PFV infection, there should also be some transcription factors similar to zinc finger protein as linker proteins which could interact with Trim28 to LTR promoter. Our data support the hypothesis that Trim28 was enriched in the PFV LTR promoter and inhibited the PFV LTR-driven gene expression to establish PFV latent infection. The role of Trim28 in antagonizing PFV replication also expands the repertoire of retroviruses that are sensitive to Trim28 regulation.

The RBCC domain located in N-terminal domain of Trim28 has been reported to function as a homo-oligomer, which is recruited by the KRAB domain to the genome [45]. Previous studies have demonstrated that the RBCC domain is required for the recruitment of Trim28 to the KRAB domain containing the zinc finger transcription factors (KRAB-ZFPs), and Trim28-binding sites are enriched in the promoter regions of KRAB-ZFPs, suggesting an auto-regulation between Trim28 and the KRAB-ZFPs [45, 46]. We also found that the RBCC domain of Trim28 was important for its interaction with Tas. Trim28 contains a RING domain, which can mediate the conjugation of proteins with ubiquitin, with small ubiquitin-like modifier (SUMO) or with the ubiquitin-like molecule IFN-stimulated protein of 15 kDa (ISG15), contributes to the biological flexibility of TRIM proteins [47]. Recently, Amina et al. reported that, in myeloid cells including microglial cells, Trim28 interacts and colocalizes with the HIV-1 transactivator Tat to promote its degradation via the proteasome pathway and to repress HIV-1 gene expression [48]. In our results, we show that RBCC domain is necessary for Trim28 interacting with Tas and destabilizing Tas through ubiquitylation pathway. And, acting mainly as a scaffold for protein complexes, Trim28 can also recruit SETDB1 and HDACs, and was found to interact with HATs p300 and histone acetyltransferase PCAF [49]. HATs p300 and histone acetyltransferase PCAF were found to interact with Tas to acetylate Tas, resulting in enhanced DNA binding ability of Tas to the virus promoters [50, 51]. Thus, Trim28 may restrict PFV replication in the multiple mechanisms. Both interaction with various proteins and post-translational modification under the coordination arrangement of Trim28 may play an important role in controlling the transactivator activity of Tas.

## Conclusion

**Fig. 6:**
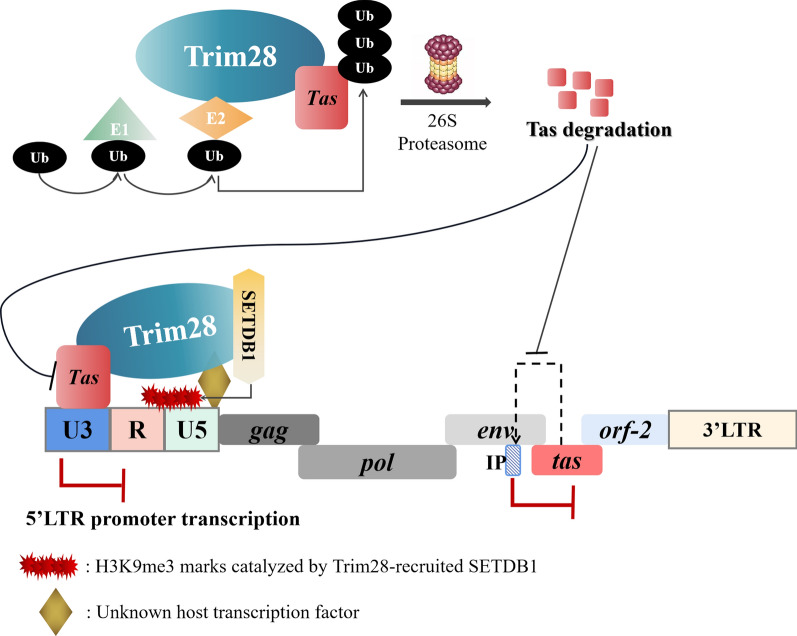
A hypothetical model depicting Trim28 mediated degradation of Tas and the regulation of PFV replication by Trim28-mediated multi-function control. Trim28 is identified as being recruited to the 5′LTR promoter region of the PFV genome. Trim28 inhibits Tas-dependent transactivation activity of PFV LTR promoter though modulating H3K9me3 marks at LTR regions. Trim28 can interact with Tas to destabilizes of Tas via ubiquitin-proteasome pathway to inhibit PFV transcription and replication

In summary (Fig. 6), we identified a cellular protein Trim28 as a novel inhibitor of PFV replication. We illustrate that the Tas-interacting protein Trim28 plays the role of a cellular restriction factor during PFV infection. Trim28, as an E3 ligase, can specifically destabilize Tas to influence its transactivation functions, and bind to PFV LTR promoter leading to the repressive H3K9me3 mark on the 5′LTR promoter of the PFV genome to inhibit PFV transcription and replication.

## Methods

### Cell culture, reagents and antibodies

All cell lines were purchased from Cell Bank of Chinese Academy of Sciences. The HEK293T and HT1080 cells were respectively grown in Dulbecco’s Modified Eagle medium (DMEM) or Minimum Essential Medium (MEM) supplemented with 10% (vol/vol) fetal bovine serum (FBS). All cell culture media and supplements were purchased from Hyclone (Hyclone Laboratories). TSA was purchased from Selleck. Anti-Myc (2276S), anti-Flag (14793S), anti-HA (3724S), anti-Trim28 (#5203) were purchased from Cell Signaling Technology. Anti-HDAC1 was purchased from Bethyl. Anti-β-actin (ab3280), anti-H3K9me3 (ab8898), and anti-Trim28 (ab10483) antibodies were obtained from Abcam. Antibody against PFV Gag was kindly provided by Professor Li Zhi, and anti-Tas was produced by immunizing rabbits with Tas and purified according to standard procedures [52]. HRP-conjugated goat anti-rabbit or goat anti-mouse secondary antibodies were from Proteintech.

### Plasmids and siRNA and transfection

Plasmids LTR-Luc, IP-Luc, Myc-Tas and TK-Tas were constructed based on the infectious pHSRV13 provirus DNA [53], which was a gift from Professor Rolf M. Flügel (German Cancer Research Center). The truncated-LTR firefly luciferase reporters (as the Fig. 2e shown) were also amplified from pHSRV13 and inserted into pGL3-Basic plasmid (Promega). pHSRV13 was also used as the template for amplifying the entire *tas* gene, and the fragment was inserted into pCMV-Myc and pEGFP-C1. Flag-Trim28 and a series of HA-tagged truncated Trim28 constructs were a gift from professor Lan Ke (Wuhan University). All the primers used for plasmids construction were listed in Table 1. Plasmid transfections were performed by using lipofectamine 2000 reagent (Life Technologies) according to the manufacturer’s instructions.

**Table 1:** Primers for PCR amplification and Quantitative real-time PCR primers

Description	Primers
qPCR-actin-F	5′-CACGATGGAGGGGCCGGACTCATC-3′
qPCR-actin-R	5′-TAAAGACCTCTATGCCAACACAGT-3′
qPCR-*gag*-F	5'-AATAGCGGGCGGGGACGACA-3'
qPCR-*gag*-R	5'-ATTGCCACGCACCCCAGAGC-3'
qPCR-*tas*-F	5'-GGAACAATCAGATACTGACCCT-3'
qPCR-*tas*-R	5'-CCAACTTCAGGATCCCATCTT-3'
ChIP-GAPDH-pro-F	5′-GAAGGTGAAGGTCGGAGTCA-3′
ChIP-GAPDH-pro-R	5′-CCCATACGACTGCAAAGACC-3′
ChIP-LTR-U3-1-F (52–187)	5′-GGGAAGGAAGTGAAGAAC-3′
ChIP-LTR-U3-1-R (52–187)	5′-TTGGATGTCAGAGGGAGT-3′
ChIP-LTR-U3-2-F(151–166)	5′-ACTCCCTCTGACATCC-3′
ChIP-LTR-U3-2-R(353–368)	5′-TTTTCGGTGTCTGTCA-3′
ChIP-LTR-U3-3-F(355–370)	5′-AAGCCACAGACAGTAA-3′
ChIP-LTR-U33-3-R(574–589)	5′-TGCATCCCACTGTTCT-3′
ChIP-LTR-U3-4-F (573–588)	5′-CACGTAGGGTGACAAG-3′
ChIP-LTR-U3-4-R (584–800)	5′-GAGAAGTGATGAGCGAC-3′
ChIP-LTR-R-F (831–955)	5′-GCTCTTCACTACTCGCTG
ChIP-LTR-R-R (831–955)	5′-GCAATCACCCTTACAATC
ChIP-LTR-U5PBS-F (1072–1167)	5′-CTTAAATGATGTAACTCCT-3′
ChIP-LTR-U5PBS-R (1072–1167)	5′-TACAAATAAACCCGACTT-3′
ChIP-IP-1-F (9019–9108)	5′-CTGGACTTTAAAAGGCCACT-3′
ChIP-IP-1-R (9019–9108)	5′-AACCAAATGTGGTAATCT-3′
ChIP-IP-2-F (9013–9196)	5′-TTTGGTTGGAATTATTGC-3′
ChIP-IP-2-R (9013–9196)	5′-AGCTTTTGCTCTTTCAAT-3′
pSuper-shTrim28-F *(target sequence)*	5′-GATCCCC*GCGTCCTGGCACTAACTCA*TTCAAG AGA*TGAGTTAGTGCCAGGACGC*TTTTTA-3′
pSuper-shTrim28-R *(target sequence)*	5′-GATCCCC*GCATGAACCCCTTGTGCTG*TTCAAG AGA*CAGCACAAGGGGTTCATGC*TTTTTA-3′
LTR-U3-F/Mlu I (pGL3-PFV-LTR-U3-luc)	5′-ATACGCGTTGTGGTGGAATGCC-3′
LTR-U3-R/Bgl II (pGL3-PFV-LTR-U3-luc)	5′-GGAAGATCTCCCGTACAAT-3′
LTR-U3-R-R/Bgl II (pGL3-PFV-LTR-U3R-luc)	5′-GGAAGATCTAGGTTCTCGAAT-3′
LTR-U3-R-U5-R/Bgl II (pGL3-PFV-LTR-U3RU5-luc)	5′-GGAAGATCTATTGTCATGGAA-3′
LTR-PBS-R/Bgl II (pGL3-PFV-LTR-U3RU5pbs-luc)	5′-GAAGATCTGCCCCACGTTG-3′

### Virus preparation and infection

HEK293T cells were transiently transfected with the pHSRV13 proviral plasmid using the PEI transfection reagent [54]. After 48 h transfection, cells and culture medium were freeze-thawed for three cycles to release viruses. To prepare virus stocks, the culture supernatant was centrifuged at 4000×*g* for 10 min and filtered through a 0.22 μm-pore-size filter membrane. HT1080 cells were infected with the PFV stock at least for 48 h to acquire PFV virions. Cells and culture medium were freeze-thawed for three cycles to release viruses. To prepare virus stocks, centrifuged at 4000×*g* for 10 min and filtered through a 0.22 μm-pore-size filter membrane and stored at − 80 °C. To assess the viral titer, HT1080 cells were seeded into 96-well plates, and the medium was replaced after 1.5 h incubation. Then, the supernatant was replaced with growth medium and cells were maintained for 48 h. Virus titers were calculated as 50% tissue culture infectious doses (TCID50) using the Reed-Münch method [55].

Cells were seeded into 6-well or 12-well plates and cultured until 80% confluency was reached. Then, the cells were infected with PFV (MOI = 0.1). After 1.5 h infection, the supernatant was replaced with growth medium and maintained (2% FBS) at 37 °C for the indicated time.

## Western blotting

For whole-cell lysates, cells were washed twice with ice-cold phosphate-buffered saline (PBS) and lysed on ice with radioimmunoprecipitation assay buffer (Beyotime Biotechnology) containing a protease inhibitor cocktail. Thereafter, the cellular lysates were cleared at 13,000 rpm for 15 min at 4 °C. Nuclear and cytoplasmic protein extracts were prepared using a Nuclear and Cytoplasmic Protein Extraction kit (Beyotime Biotechnology). The samples were boiled at 100 °C for 10 min with sample loading buffer (5% SDS, 10% glycerol, 60 mM Tris pH 6.8, 5% β-mercaptoethanol, and 0.01% bromophenol blue). The protein samples were resolved by sodium dodecyl sulfate–polyacrylamide gel electrophoresis (SDS-PAGE) and transferred to polyvinylidene fluoride (PVDF) membranes. The membranes were blocked in 5% nonfat milk-TBST for 3 h at room temperature and incubated with primary antibodies overnight at 4 °C followed. Bound antibodies were visualized by horseradish peroxidase-conjugated secondary antibody (Sungene Biotech) for 1.5 h at room temperature and enhanced chemiluminescence (ECL) system (Advansta) with a Kodak imager (Carestream Health). The quantitative analysis of the relative intensities of proteins (normalized to β-actin) was performed with Quantity One Software (Bio-Rad) and GraphPad Prism 5. All data are representative of three independent experiments with triplicate samples. Statistical significance was analyzed with Student’s *t-*test. All experiments in this study are repeated at least for three times.

### Real-time quantitative PCR

Cells treated with various stimuli were harvested in TRIzol (Invitrogen) and first-strand cDNA was synthesized with Revert AidTM First Strand cDNA Synthesis Kit (Thermo Scientific) according to the manufacturer’s protocol. Gene expression was examined with a SYBR green Real-Time PCR master mix kit (Toyobo) according to the manufacturer’s protocol. Values for the relative quantification were calculated by the 2^−△△Ct^ method. Melting curve analysis was performed to verify the specificity of the products, and each sample was tested in triplicate. All primers are listed in Table 1. Quantification of β-actin transcripts was used to normalize RNA amounts. All data are representative of three independent experiments with triplicate samples. Statistical significance was analyzed with a Student’s *t-*test. (*p < 0.05, **p < 0.01, ***p < *0.001*).

### Luciferase reporter assay

HEK293T cells (4 × 10^4^) were cultured in 24-well plates and transfected with the pGL3-PFV-LTR-luc or pGL3-PFV-IP-luc reporter plasmids and a Renilla luciferase reporter plasmid (pRL-TK, Promega) as an internal control. The empty vector was used to equalize the total amount of DNA. Twenty-four hours after transfection, the cells were lysed in passive lysis buffer, and the firefly and Renilla luciferase activities were performed using the Dual-Luciferase Reporter Assay System following the manufacturer’s protocol (Promega). All experiments were performed in triplicate. The firefly luciferase activity was normalized on the Renilla luciferase activity and expressed as the fold change relative to the activity in the vector-transfected cells. Data represent the average of three independent experiments, and error bars represent SD.

### Foamy virus activated luciferase (FAL) assay

For the foamy virus activated luciferase (FAL) assay, HEK293T cells were transfected with pCMV-Flag-Trim28 for 24 h (pCMV-Flag was transfected as an negative control), and the cells were infected with PFV (MOI = 0.1) for another 24 h, then those infected HEK293T were incubated with a PFV indicator cell line (BHK21-derived indicator cells containing a luciferase gene under the control of the PFV LTR) for 48 h to detecting PFV viral load, and RL-TK plasmid expressing Renilla (RLu) luciferase was transfected into PFV indicator cell line as an internal control 12 h before incubation.

### Indirect immunofluorescence

Briefly, cells were plated on cover slides in 24-well plates and allowed to settle overnight. Cells were transfected with pEGFP-N1-Tas for 48 h. Following transfection, the cells were permeabilized with 0.5% Triton X-100 (Thermo Fisher) for 15 min on ice and then fixed in 4% paraformaldehyde (Santa Cruz) for 20 min. Cells were incubated with the primary anti-Trim28 antibody (1:1000, Abcam ab10483) at 4 °C for the night and then a Cy3-conjugated goat anti-rabbit IgG (1:100, Proteintech) at 37 °C for 1 h. 4′, 6-Diamidino-2-phenylindole (DAPI; Life Technologies) was used to stain the nuclei. Coverslips were inverted onto slides containing 50% glycerol, and fluorescence signals were visualized with a confocal fluorescence microscope (Leica-LCS-SP8-STED, Medical research for structural biology of Basic Medical Sciences, Wuhan University).

### Co-immunoprecipitation

In brief, cells were lysed in Nonidet P-40 lysis buffer containing 150 mM NaCl, 1 mM EDTA, 1% Nonidet P-40, and 1% protease and phosphatase inhibitor cocktail (Beyotime Biotechnology). The respective proteins were immunoprecipitated with IgG (control) or the specific antibodies, Protein A/G Plus-Agarose (Santa Cruz Biotechnology, sc-2003) and the precipitants were washed three times with a high salt lysis buffer containing 500 mM NaCl, followed by immunoblot analysis. The antibodies were diluted in 3–5% (wt/vol) fat-free milk (BD Biosciences) or 3% BSA (Sigma) in TBS (1:500–1:2000).

## ChIP

ChIP assays were performed as previously Wu’s lab described [56]. In briefly, cells were fixed with 1% formaldehyde for 10 min and quenched by 0.125 M glycine for 5 min at room temperature. After cross-linking, the cells were washed three times with PBS and then harvested in ChIP lysis buffer (50 mM Tris–HCl pH 8.0, 1% SDS, 5 mM EDTA) followed by sonication to fragment the DNA to 400–600 bp. The lysate was centrifuged at 4 °C for 15 min and ChIP dilution buffer (20 mM Tris–HCl, pH 8.0, 150 mM NaCl, 2 mM EDTA, 1% Triton X-100) was added to the supernatant (4:1 volume). The resulting lysate was then incubated with protein G beads and antibodies at 4 °C overnight. The beads were washed five times and DNA was eluted in ChIP elution buffer (0.1 M NaHCO_3_, 1% SDS and 30 μg/ml proteinase K). The elution was incubated at 65 °C overnight and DNA was extracted with a DNA purification kit (Tiangen). The purified DNA was assayed by quantitative PCR with a SYBR green Real-Time PCR master mix kit (Toyobo). The primer information is listed in Table 1. Data shown are mean ± standard deviations (SD) of representative experiments. At least three biological replicates were analyzed in each experiment. A *t-*test was used for statistical analysis.

### Cellular ubiquitination assay

A total of 1 × 10^7^ HEK293T cells were seeded in 10 cm dish and then were transfected with HA-tagged ubiquitin (HA-Ub) (4 μg), Myc-Tas(8 μg), with Flag-Trim28 (8 μg) or pCMV-Flag (8 μg) as a negative control. After 24 h, the cells were washed with ice-cold phosphate-buffered saline and collected with 1 mL IP lysis buffer (Beyotime) with 1 mM PMSF and lysed at 4 °C for 30 min. Soluble protein fraction was separated by centrifugation at 12,000 rpm for 10 min, and 1 mg whole cell protein was immunoprecipitated with 2 μg anti-Myc antibody for Myc-Tas, for 4 h and then incubated with 40 μL Protein A/G plus-Agarose per sample overnight. The beads were washed with IP buffer 5 times and resuspended in 20 μL lysis buffer and 20 μL 2 × SDS loading buffer and boiled for 5 min. The immunoprecipitate was analyzed with anti-HA antibody by Western blot. The whole cell lysate was assessed with anti-Flag, and anti-myc by Western blot.

### Statistical analysis

Data were expressed as the means ± SD. Statistical analyses were performed using GraphPad Prism to evaluate the differences between experimental groups. Statistical significance was determined using Student’s *t-*test and expressed as p-values. *p < *0.05* was considered to be statistically significant. (*p < 0.05, **p < 0.01, ***p < *0.001*).

## Data Availability

The original contributions presented in the study are included in the article. Further inquiries can be directed to the corresponding author.

## Abbreviations

PFV: Prototype foamy virus
APOBEC3: Apolipoprotein B mRNA-editing enzyme, catalytic polypeptide-like 3
LTR: Long terminal repeat
IP: Internal promoter
Trim28: The tripartite motif-containing protein 28
H3K9me3: Histone H3 containing the trimethylated lysine 9
SETDB1: SET domain bifurcated 1
MOI: Multiplicity of infection
GFP: Green fluorescent protein
ChIP: Chromatin immunoprecipitation assay
DMSO: Dimethyl sulfoxide
HIV-1: Human immunodeficiency virus type 1
HTLV: Human T-cell leukemia virus
